# The future of a partially effective HIV vaccine: assessing limitations at the population level

**DOI:** 10.1007/s00038-019-01234-z

**Published:** 2019-04-13

**Authors:** Christian Selinger, Dobromir T. Dimitrov, Philip A. Welkhoff, Anna Bershteyn

**Affiliations:** 1 Institute for Disease Modeling, 3150 139th Ave SE, Bellevue, WA 98005 USA; 2 Fred Hutchinson Cancer Research Center, 1100 Fairview Ave N, Seattle, WA 98109 USA

**Keywords:** HIV vaccine, Epidemiological modeling, South Africa, Product development

## Abstract

**Objectives:**

Mathematical models have unanimously predicted that a first-generation HIV vaccine would be useful and cost-effective to roll out, but that its overall impact would be insufficient to reverse the epidemic. Here, we explore what factors contribute most to limiting the impact of such a vaccine.

**Methods:**

Ranging from a theoretical ideal to a more realistic regimen, mirroring the one used in the currently ongoing trial in South Africa (HVTN 702), we model a nested hierarchy of vaccine attributes such as speed of scale-up, efficacy, durability, and return rates for booster doses.

**Results:**

The predominant reasons leading to a substantial loss of vaccine impact on the HIV epidemic are the time required to scale up mass vaccination, limited durability, and waning of efficacy.

**Conclusions:**

A first-generation partially effective vaccine would primarily serve as an intermediate milestone, furnishing correlates of immunity and platforms that could serve to accelerate future development of a highly effective, durable, and scalable next-generation vaccine capable of reversing the HIV epidemic.

**Electronic supplementary material:**

The online version of this article (10.1007/s00038-019-01234-z) contains supplementary material, which is available to authorized users.

## Introduction

An estimated 2.1 million people were infected with HIV in 2015 (Unaids 2016). Despite increasing numbers of people on antiretroviral treatment (ART), there is still a need to scale up HIV prevention in order to counter the global epidemic on a population level. Existing prevention modalities such as condoms, medical male circumcision, treatment as prevention, and oral pre-exposure prophylaxis (PrEP) face limitations such as negotiability, stigma, access, adherence, retention, and efficacy (Pettifor et al. 2013; Celum et al. 2015). A breakthrough in HIV prevention such as a highly effective vaccine is urgently needed. The Pox-Protein Public-Private Partnership (P5) is working to build on the findings of the RV144 trial (Russell and Marovich 2016) with a currently ongoing Phase 2b/3 trial (HVTN 702) in South Africa (ClinicalTrails 2017). The vaccine tested in the Thailand-based RV 144 trial consisted of one prime dose (canarypox vector with *gag, pol* and *env* HIV genes) followed by two prime–boost doses (adding the viral protein *gp120*). Modified intent-to-treat analysis showed marginal efficacy of $\sim 30\%$ protection against heterosexual HIV acquisition. HTVN 702 builds on RV 144 by utilizing the same vector with HIV genes specific to the clade predominantly circulating in South Africa, a different adjuvant and an extended regimen of five doses. With its complex immunization schedule and anticipated waning of immunity, the regimen is likely to provide only partial efficacy over limited time. Such vaccine could be seen as a first-generation product that must still be improved upon in order to fundamentally transform the HIV epidemic. Mathematical models (Medlock et al. 2017; Hontelez et al. 2011; Smith et al. 2016; Andersson and Stover 2011; Harmon et al. 2016; Moodley et al. 2016a, b; Hankins et al. 2011; Phillips et al. 2014; Adamson et al. 2017; de Montigny et al. 2018) suggest that the first-generation vaccine would be useful and cost-effective to roll out, but that its overall impact will be modest. Here, we explore what factors contribute collectively to limiting the impact of such a vaccine.

## Methods

Motivated by the HIV vaccine trial HVTN 702 currently ongoing in South Africa, we developed an agent-based model of the HIV epidemic in the South African population to forecast HIV infections over a 20-year time horizon, from year 2027 to 2047. The choice of this time horizon was based on the originally planned primary completion date of the vaccine trial in 2021 (ClinicalTrails 2017) and delays in its start. Thus, the year 2027 was assumed as plausible earliest possible rollout of mass vaccination. As compared to a reference case with no HIV vaccine, we evaluate implementation of strategies for initiation of HIV vaccination in a population aged between 18 and 34.

### Model setup and calibration

We modified EMOD-HIV v2.5, an age-stratified and individual-based network model of HIV of South Africa, to incorporate HIV vaccination according to pox-protein HIV vaccine regimens (such as the regimen currently being tested in HVTN 702). Because EMOD is an individual-based model, interventions such as a time-varying course of vaccine efficacy can be applied to each individual according to his or her own timing of vaccination and adherence to the booster series. This renders the model well suited for a nuanced analysis of the anticipated time-dependent efficacy of the pox-protein HIV vaccine regimen.

The parameters, model input values, sources, projections, and sensitivities of the epidemic projection without vaccine, used as the reference group for comparison, have been described previously (Bershteyn et al. 2012, 2013; Klein et al. 2014; Selinger et al. 2019). A detailed model description, default parameters, user tutorials, model installer, and source code are available for download at http://idmod.org/software. EMOD-HIV is an individual-based model that simulates transmission of HIV using an explicitly defined network of heterosexual relationships that are formed and dissolved according to age- and risk-dependent preference patterns (Klein 2012). The synthetic population was initiated in 1960, and population recruitment and mortality were assumed to be proportional following age- and gender-stratified fertility and mortality tables and projections from the 2012 UN World Population Prospects (UnitedNations 2016). Since the population size of South Africa exceeds the computational limit of simulated agents, we assumed that one simulated agent corresponds to 300 real-world individuals. The model was calibrated to match retrospective estimates of age- and gender-stratified, national-level prevalence, incidence, ART coverage and all-cause mortality from nationally representative HIV surveys in South Africa (Shisana et al. 2010, 2014; Eaton et al. 2015; Rehle et al. 2007; Shisana and Simbayi 2002) and demographic data (Statistics South Africa 2014) (see Figure 1 in Supplementary Materials for an overlay between model calibration outputs and source data). We also refer to a comparative calibration study comprising ten models (including EMOD) of HIV transmission in South Africa (see supplementary material in Eaton et al. 2015). For the purpose of this modeling study, we emphasized calibration of model parameters concerning risk assortativity during partnership formation, duration, and concurrency of partnerships as well as condom usage. For each simulated vaccine scenario, we used the 50 most likely parameter sets obtained from the gradient-descent-based calibration process (see Table 1 in Supplementary Material). The age patterns of sexual mixing were configured to match those observed in the rural, HIV-hyperendemic province of KwaZulu-Natal, South Africa (Ott et al. 2011). Recently, a validation study showed that self-reported partner ages in this setting are relatively accurate, with 72% of self-reported estimates falling within 2 years of the partners' actual date of birth (Harling et al. 2015). In addition, a recent field study on age-mixing patterns in Cape Town came to similar conclusions concerning the age gap between men and women (Beauclair et al. 2018), suggesting that the age-mixing data used in our model is not specific to KwaZulu-Natal but applies more generally to South Africa. Further, the transmission patterns observed in EMOD (Bershteyn et al. 2013) are consistent with those revealed in a recent phylogenetic analysis of the age/gender patterns of HIV transmission in this setting (de Oliveira et al. 2017).

Transmission rates within relationships depend on HIV disease stage, male circumcision, condom usage, coinfections. Viral suppression achieved through antiretroviral therapy (Attia et al. 2009; Marconi et al. 2011) is assumed to reduce transmission by 92%—an estimate based on observational data of serodiscordant couples in which outside partnerships could have contributed to HIV acquisition (Donnell et al. 2010).

### HIV treatment and prevention

We configured the EMOD healthcare system module to follow trends in antiretroviral therapy (ART) expansion in South Africa. Treatment begins with voluntary counseling and testing (VCT), antenatal and infant testing, symptom-driven testing, and low level of couples testing. The model includes loss to follow-up between diagnosis and staging, between staging and linkage to ART or pre-ART care, and during ART or pre-ART care (Mugglin et al. 2013). Projections of South Africa treatment expansion in the no-vaccine reference group are calibrated to reflect a gradual decline of HIV incidence without elimination, so that HIV remains endemic through 2050 (Eaton et al. 2013). All scenarios included medical male circumcision (Connolly et al. 2008) at 22% coverage and conferring 60% reduction in acquisition risk with lifetime durability. Condom usage was dependent on four relationship types (transitory, informal, marital, and commercial), with per act usage probability ramping up to median values of 62%, 39%, 26%, and 85% by 2027 across parameter draws. To allow for maximum hypothetical impact of a vaccine, we assume proportion of HIV-positive adults diagnosed of about 85% and future ART coverage of about 60% [consistent with the current guidelines assumed in the HIV/TB Investment Case Report for South Africa (South African National AIDS Council 2016), see also (Johnson et al. 2017b)] and no use of oral PrEP. Variations on these assumptions, explored elsewhere (Selinger et al. 2017, 2019), only further diminish the impact of the vaccine.

### HIV vaccine efficacy

We incorporated a parametric model of time-dependent vaccine efficacy that was hypothesized for the pox-protein regimen based on results from RV144. We included the time series of efficacy associated with each of the originally planned five doses administered during the study and possible booster doses (described in detail below) beyond the 24-month duration of the first stage of the study. Specifically, the pox-protein dosing schedule that was modeled consisted of a series of five immunizations over 12 months. ALVAC-HIV-C was administered at months 0 and 1, followed by ALVAC-HIV-C+gp120 at months 3, 6, and 12. The protocol was later revised to include a boost at month 18, but this was not considered at the time of this modeling effort. Time-dependent vaccine efficacy was interpreted as a per exposure reduction in the probability of acquisition parameterized by an impulse and exponential decay model (Selinger et al. 2019).

$$\begin{aligned}&\text {TimeDependentVaccineEfficacy}(t)\\&\quad :=\sum _{i\in \text {Schedule}, i\le t} (a_i+b_i)\hbox{e}^{-\omega (t-i+d)} \end{aligned}$$

where $a_i$ is the efficacy increase of immunization with ALVAC-HIV-C, $b_i$ is efficacy increase after ALVAC-HIV-C + gp120 immunization, $\omega$ is the efficacy decay rate per month, and *d* is the delay between immunization and initiation of protective effect in months.

Assuming uniformly distributed exposure over a given time span in the trial, we calculated the cumulative vaccine efficacy (corresponding to the efficacy estimate from the trial) as the area under the curve of the instantaneous vaccine efficacy rescaled by the length of the time span.

$$\begin{aligned}&\text {VaccineEfficacy}(t)\\&\quad := \frac{1}{t}\int _0^t\text {TimeDependentVaccineEfficacy}(s)\hbox{d}s \end{aligned}$$

In anticipation of efficacy results for HVTN 702, we modeled time-dependent vaccine efficacy based on results from statistical models (Robb et al. 2012) for RV144 study outcomes using a point estimate of 58% shortly after the month 6 vaccination and cumulative efficacy of 31.2% over 42 months. We adjusted the parameters of the efficacy function such that the cumulative vaccine efficacy over 24 months after the first dose is 50% and obtained values $a_i=0.08$, $b_i=0.34$, $\omega =0.065$ and $d=0.1$.

### Booster schedule and efficacy

For the purpose of model projections beyond the primary trial endpoint, we also implemented up to four two-yearly boosters starting at month 36 with fixed return rates of 100, 80, or 50% per booster to cover a total of 10 years of vaccine efficacy. We assumed booster efficacy to follow the same parameterization as ALVAC-HIV-C + gp120 doses from the primary immunization series during the first 12 months. Booster eligibility depended on having received the primary immunization series or the booster previously. Missing a booster resulted in loss of eligibility for subsequent boosters, which may or may not prove to be the case when the vaccine is implemented. Individuals who tested HIV positive were not eligible for future boosters. We assumed that four booster doses after the primary first-year series were necessary to confer one decade of protection. Booster eligibility was limited by the target age range of catch-up vaccination (see below).

### Nested hierarchy of vaccination

Because of the nested sequence of steps that are required for a partially effective vaccine to confer benefit, we conceptualized the steps as a cascade of prevention (Garnett et al. 2016), analogous to the “cascade of care” in which an individual must be diagnosed, initiated on treatment, and achieving viral suppression in order to maximally benefit from HIV care and treatment (Johnson et al. 2017b). We simulated a series of vaccination scenarios, sequentially incorporating different limitations of the vaccine and its rollout (see Table 1). For each limitation, we quantified the decline in population-level impact, as measured by the percent reduction in cumulative new infections relative to a base case scenario without vaccine between 2027 and 2047 for individuals aged 15–49. Since we did assume neither scale-up of ART coverage nor use of oral pre-exposure prophylaxis in the time period under investigation, percent reduction in new infections represents an upper bound relative to the maximum number of new infections prevented; i.e., more optimistic assumptions on ART scale-up would result in less infections prevented.

In all simulation scenarios, we assumed an initial catch-up vaccination campaign starting in 2027, where all men and women aged between 18 and 34 would be vaccinated, followed by vaccination of all 18-year-olds in subsequent years until 2047. The scenarios differed by the time it would take to scale up catch-up vaccination, and also by coverage, durability, and efficacy of the vaccine. First, we considered an idealized vaccine, providing complete protection (indefinite, 100% efficacy) without waning and available for use in 2027, even though we recognize the impracticality of such a scenario. Second, we simulated a more gradual vaccination, assuming a 5-year linear scale-up of catch-up vaccination starting in 2027 before reaching full coverage by 2032, from which on 18-year-olds are vaccinated in subsequent years until 2047. Third, we considered a vaccine with limited duration of complete protection, assuming 10 years of full efficacy (100%), after which vaccine recipients were no longer protected. Next, we simulated a vaccine with partial efficacy that varied over 10-year protection period, averaging 50% over the first 2 years (including the first year of intensive vaccination) and falling to 15–30% over the next 8 years depending on booster frequency. Finally, we considered a wide range of hypothetical coverage levels of 60, 30, and 10% at various return rates for booster vaccination. We chose a population aged 15–49 as reference group to cover the sexually active population and vaccination age between 18 and 34 to target the core of the population at risk. The age range used for vaccination in our model is the same as in the currently ongoing vaccine trial HVTN 702. Adolescent vaccination would require further licensure procedures and potentially delay vaccination scale-up and was therefore excluded. The impact of age- and gender-dependent targeting for this vaccine regimen was explored elsewhere (Selinger et al. 2019).

**Table 1:** Nested hierarchy of vaccination scenarios simulated for South Africa over the years 2027 through 2047

Scenario	Coverage (%)	Scale-up (years)	Efficacy (%)	Durability (years)	Waning	Return rate for booster (%)
1	100	0	100	20	No	NA
2	100	5	100	20	No	NA
3	100	5	100	10	No	NA
4	100	5	50	10	Yes	100
5	60	5	50	10	Yes	100
6	30	5	50	10	Yes	100
7	10	5	50	10	Yes	100
8	100	5	50	10	Yes	80
9	60	5	50	10	Yes	80
10	30	5	50	10	Yes	80
11	10	5	50	10	Yes	80
12	100	5	50	10	Yes	50
13	60	5	50	10	Yes	50
14	30	5	50	10	Yes	50
15	10	5	50	10	Yes	50

## Results

**Fig. 1:**
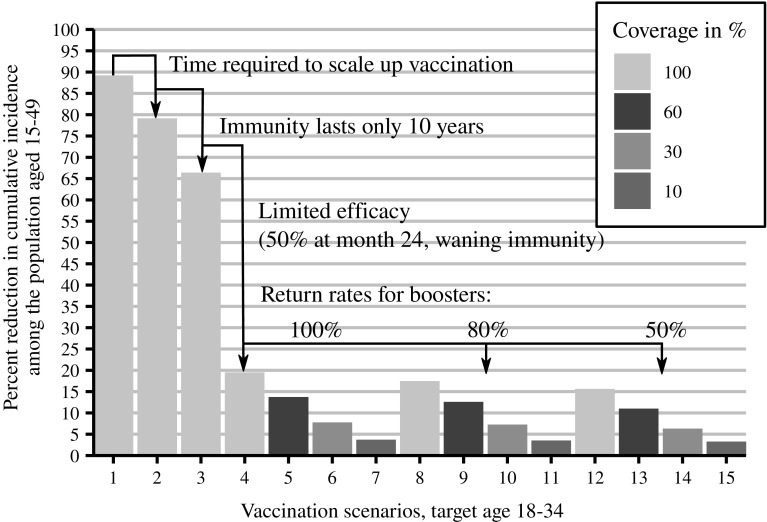
Percent reduction in cumulative new infections in a nested hierarchy of HIV vaccination scenarios. The percent values refer to the cumulative number of infections prevented by vaccination in South Africa between 2027 and 2047 in populations aged 15–49, divided by the cumulative number of infections in non-vaccine reference simulations for the same time period and age range

Modeling results (see Fig. 1) suggest that an ideal vaccine available in 2027 and covering all 18–34-year-olds could prevent 89% of all infections occurring in the South African population aged 15–49 over the 2027–2047 interval. A more realistic 5-year scale-up to reach full coverage entails a modest drop in impact to 79%, while limiting protection durability to 10 years results in a further drop to 66% infections averted. The second large reduction in intervention impact (from 66% to 20%) is due to assumptions of partial and waning efficacy. In total, factors related to vaccine durability, scale-up, and efficacy result in a difference of 69% in vaccination effectiveness. Decreasing coverage from 100 to 60% will further attenuate the epidemic impact to just above 14% while problems with retention to the series of boosters will prevent as few as 11% of cumulative infections. In our most pessimistic scenario, assuming 10% coverage and 50% booster return rate, the epidemic impact drops down to 3% of infections averted.

Another way to visualize the implications of the simulated vaccination scenarios is considering the epidemic curves, i.e., average annual number of new infections (see Figure 2 in Supplementary Materials). Starting out with an estimated 362,000 new infections in 2017, the first two scenarios with vaccination in 2027 at hypothetical 100% efficacy would lead to less than 1000 new infections in 2047. Limited durability of the same vaccine increases new annual infections to 96,000 and efficacy assumptions of 50% with waning results in 260,000 new infections in 2047. Achieving high return rates for booster vaccination produces little change in the epidemic curve.

## Discussion

This modeling analysis of a nested hierarchy of vaccination clearly highlights three dominant reasons for the limited population-level impact of a partially effective HIV vaccine, namely the time required to scale up mass vaccination, limited durability and waning of efficacy. Each of these obstacles poses particular challenges for public health implementation. First, fast and high uptake of an HIV vaccine in South Africa will be difficult to achieve in an adult catch-up population, compared to the success of the school-based two-dose Human Papillomavirus (HPV) Vaccination Program (Delany-Moretlwe et al. 2018). In addition to logistic challenges for vaccine manufacturers and healthcare systems, acceptance for a newly introduced vaccine within a short period of time may prove crucial. Indeed, research in social psychology stresses that HIV testing, HIV stigma, mistrust of the healthcare system, and concerns about sexual disinhibition may be barriers to vaccine uptake in young adults, with noticeable differences between men and women (Sayles et al. 2009; Rubincam et al. 2018). Second, limited durability raises the questions whether revaccination with a complex regimen 10 years after the initial vaccination can be implemented at high coverage, given the challenges of achieving high vaccination rates in adults (CDC 2016). Finally, partial efficacy and waning remain the strongest factors limiting impact at the population level.

We simulated 50% efficacy at month 24 as an illustration, but we hope that efficacy in HVTN 702 will be higher due to the modified regimen compared to RV144. From a public health perspective, the rollout of a partially effective, quickly waning vaccine must be considered with caution and as an addition to existing HIV prevention measures, as it is only a first step toward vaccine development with improved immunogenicity.

The present modeling study bears several limitations. The uncertainty in the number of new infections when reaching the prospective start of mass vaccination in 2027 is a major one. Although recently published numbers of 270,000 (95% CI 240,000–300,000) new infections in South Africa in 2017 (Unaids 2018) are in line with our simulation results of 362,000 new infections (bearing in mind the more pessimistic assumptions on ART scale-up in our model), it remains difficult to anticipate dynamics of the HIV epidemic over a longer timescale. Another limitation stems from the fact that in South Africa, the HIV epidemic is highly heterogeneous between provinces, mostly differing in prevalence of male circumcision and non-marital relationships (Johnson et al. 2017a). In this context, national-level models (such as the present study) must be utilized with caution; e.g., it has been shown that spatial heterogeneity and associated migration strongly influence HIV transmission  (Bershteyn et al. 2018; Dobra et al. 2017; Coffee et al. 2007; Voeten et al. 2009) and therefore vaccination implementation. Considering constrained vaccine coverage regarding heterogeneously distributed geospatial and behavioral risk would raise ethical questions of how to devise vaccine rollout in an optimal way  (Keeling and Shattock 2012). At last, we would like to mention the likely impact of risk compensation (Eaton and Kalichman 2007; MacPhail et al. 2012). Increases in risky behavior by vaccine recipients and their partners such as reductions in condom use and an increase in multiple partners potentially impair population-level vaccine impact, especially for a partially effective vaccine.

Our analysis suggests that optimizing the efficacy of a broadly used vaccine should be a continuous process because of its critical contribution to the vaccination impact. Maximizing coverage has been rightly in the focus of all effectiveness analyses since it is likely to pose a challenge for a vaccine candidate with such a complex and lengthy dosing schedule. However, its ability to improve vaccination impact is already limited by the speed of vaccine rollout and imperfect efficacy. Timely scale-up of manufacturing capacities, improved immunogenicity, and reassessing risk–benefit considerations for target populations with high-risk profile during the licensure process could help to overcome the major obstacles to population-level impact identified in this analysis.

Model-based estimates of the impact of pox-protein-like vaccines are often met with disappointment. Several modeling studies have explicitly considered waning efficacy in a South African context and concluded that only frequent revaccination at high coverage would yield proportions of infections prevented appreciably high at about 15% (Andersson and Stover 2011; Hontelez et al. 2011). Our modeling study contributes to the existing body of evidence in several ways. First, it is based on the currently ongoing HTVN 702 trial (more realistic start of vaccine rollout, a more detailed immunization schedule). Second, our model is unique in that it is an age-structured agent-based network model, such that secondary benefits of vaccination within sexual contact networks can also be factored in. Third, it explores systematically possible shortcomings of the vaccine.

This analysis aims to clarify why the absolute impact of a partially effective multi-dose vaccine with limited durability is likely to be modest. Though worthwhile to develop and make available, such a vaccine is unlikely to reverse the course of the HIV epidemic. Rather, a first-generation partially effective vaccine would primarily serve as an intermediate milestone, furnishing correlates of immunity and platforms that could serve to accelerate future development of a highly effective, durable, and scalable next-generation vaccine capable of reversing the HIV epidemic.

## Electronic supplementary material

Below is the link to the electronic supplementary material.

Supplementary material 1 (pdf 618 KB)

## References

[CR1] Adamson B, Dimitrov D, Devine B, Barnabas R (2017). The potential cost-effectiveness of HIV vaccines: a systematic review. Pharmacoecon Open.

[CR2] Andersson KM, Stover J (2011). The potential impact of a moderately effective HIV vaccine with rapidly waning protection in South Africa and Thailand. Vaccine.

[CR3] Attia S, Egger M, Müller M, Zwahlen M, Low N (2009). Sexual transmission of HIV according to viral load and antiretroviral therapy: systematic review and meta-analysis. AIDS.

[CR4] Beauclair R, Hens N, Delva W (2018). The role of age-mixing patterns in HIV transmission dynamics: novel hypotheses from a field study in Cape Town, South Africa. Epidemics.

[CR5] Bershteyn A, Klein DJ, Wenger E, Eckhoff PA (2012) Description of the EMOD-HIV Model v0.7. ArXiv e-prints 1206.3720

[CR6] Bershteyn A, Klein DJ, Eckhoff PA (2013). Age-dependent partnering and the HIV transmission chain: a microsimulation analysis. J R Soc Interface.

[CR7] Bershteyn A, Mutai KK, Akullian AN, Klein DJ, Jewell BL, Mwalili SM (2018). The influence of mobility among high-risk populations on HIV transmission in Western Kenya. Infect Dis Model.

[CR8] CDC (2016) Vaccination coverage among adults in the United States, National Health Interview Survey. https://www.cdc.gov/vaccines/imz-managers/coverage/adultvaxview/pubs-resources/NHIS-2016.html

[CR9] Celum CL, Delany-Moretlwe S, McConnell M, van Rooyen H, Bekker LG, Kurth A, Bukusi E, Desmond C, Morton J, Baeten JM (2015). Rethinking HIV prevention to prepare for oral PrEP implementation for young African women. J Int AIDS Soc.

[CR10] ClinicalTrails (2017) Pivotal phase 2b/3 ALVAC/bivalent gp120/MF59 HIV vaccine prevention safety and efficacy study in South Africa. https://clinicaltrials.gov/ct2/show/NCT02968849. Accessed 2017-03-31

[CR11] Coffee M, Lurie MN, Garnett GP (2007). Modelling the impact of migration on the HIV epidemic in South Africa. AIDS.

[CR12] Connolly C, Simbayi LC, Shanmugam R, Nqeketo A (2008). Male circumcision and its relationship to HIV infection in South Africa: results of a national survey in 2002. S Afr Med J.

[CR13] de Montigny S, Adamson BJS, Mâsse BR, Garrison LP, Kublin JG, Gilbert PB, Dimitrov DT (2018). Projected effectiveness and added value of HIV vaccination campaigns in South Africa: a modeling study. Sci Rep.

[CR14] de Oliveira T, Kharsany ABM, Gräf T, Cawood C, Khanyile D, Grobler A, Puren A, Madurai S, Baxter C, Karim QA, Karim SSA (2017). Transmission networks and risk of HIV infection in KwaZulu-Natal, South Africa: a community-wide phylogenetic study. Lancet HIV.

[CR15] Delany-Moretlwe S, Kelley KF, James S, Scorgie F, Subedar H, Dlamini NR, Pillay Y, Naidoo N, Chikandiwa A, Rees H (2018). Human papillomavirus vaccine introduction in South Africa: implementation lessons from an evaluation of the national school-based vaccination campaign. Glob Health Sci Pract.

[CR16] Dobra A, Bärnighausen T, Vandormael A, Tanser F (2017). Space-time migration patterns and risk of HIV acquisition in rural South Africa. AIDS.

[CR17] Donnell D, Baeten JM, Kiarie J, Thomas KK, Stevens W, Cohen CR, McIntyre J, Lingappa JR, Celum C (2010). Heterosexual HIV-1 transmission after initiation of antiretroviral therapy: a prospective cohort analysis. Lancet.

[CR18] Eaton LA, Kalichman S (2007). Risk compensation in HIV prevention: implications for vaccines, microbicides, and other biomedical HIV prevention technologies. Curr HIV/AIDS Rep.

[CR19] Eaton JW, Menzies NA, Stover J, Cambiano V, Chindelevitch L, Cori A, Hontelez JAC, Humair S, Kerr CC, Klein DJ, Mishra S, Mitchell KM, Nichols BE, Vickerman P, Bakker R, Brnighausen T, Bershteyn A, Bloom DE, Boily MC, Chang ST, Cohen T, Dodd PJ, Fraser C, Gopalappa C, Lundgren J, Martin NK, Mikkelsen E, Mountain E, Pham QD, Pickles M, Phillips A, Platt L, Pretorius C, Prudden HJ, Salomon JA, van de Vijver DAMC, de Vlas SJ, Wagner BG, White RG, Wilson DP, Zhang L, Blandford J, Meyer-Rath G, Remme M, Revill P, Sangrujee N, Terris-Prestholt F, Doherty M, Shaffer N, Easterbrook PJ, Hirnschall G, Hallett TB (2013). Health benefits, costs, and cost-effectiveness of earlier eligibility for adult antiretroviral therapy and expanded treatment coverage: a combined analysis of 12 mathematical models. Lancet Glob Health.

[CR20] Eaton JW, Bacar N, Bershteyn A, Cambiano V, Cori A, Dorrington RE, Fraser C, Gopalappa C, Hontelez JAC, Johnson LF, Klein DJ, Phillips AN, Pretorius C, Stover J, Rehle TM, Hallett TB (2015). Assessment of epidemic projections using recent HIV survey data in South Africa: a validation analysis of ten mathematical models of HIV epidemiology in the antiretroviral therapy era. Lancet Glob Health.

[CR21] Garnett GP, Hallett TB, Takaruza A, Hargreaves J, Rhead R, Warren M, Nyamukapa C, Gregson S (2016). Providing a conceptual framework for HIV prevention cascades and assessing feasibility of empirical measurement with data from east Zimbabwe: a case study. Lancet HIV.

[CR22] Hankins CA, Glasser JW, Chen RT (2011). Modeling the impact of RV144-like vaccines on HIV transmission. Vaccine.

[CR23] Harling G, Tanser F, Mutevedzi T, Bärnighausen T (2015). Assessing the validity of respondents’ reports of their partners’ ages in a rural South African population-based cohort. BMJ Open.

[CR24] Harmon TM, Fisher KA, McGlynn MG, Stover J, Warren MJ, Teng Y, Nveke A (2016). Exploring the potential health impact and cost-effectiveness of AIDS vaccine within a comprehensive HIV/AIDS response in low- and middle-income countries. PLoS ONE.

[CR25] Hontelez JAC, Nagelkerke N, Bärnighausen T, Bakker R, Tanser F, Newell ML, Lurie MN, Baltussen R, de Vlas SJ (2011). The potential impact of RV144-like vaccines in rural South Africa: a study using the STDSIM microsimulation model. Vaccine.

[CR26] Johnson LF, Dorrington RE, Moolla H (2017). HIV epidemic drivers in South Africa: a model-based evaluation of factors accounting for inter-provincial differences in HIV prevalence and incidence trends. S Afr J HIV Med.

[CR27] Johnson LF, Dorrington RE, Moolla H (2017). Progress towards the 2020 targets for HIV diagnosis and antiretroviral treatment in South Africa. S Afr J HIV Med.

[CR28] Keeling MJ, Shattock A (2012). Optimal but unequitable prophylactic distribution of vaccine. Epidemics.

[CR29] Klein DJ (2012) Relationship formation and flow control algorithms for generating age-structured networks in HIV modeling. In: 2012 IEEE 51st IEEE conference on decision and control (CDC). IEEE. 10.1109/cdc.2012.6426573

[CR30] Klein DJ, Bershteyn A, Eckhoff PA (2014). Dropout and re-enrollment: implications for epidemiological projections of treatment programs. AIDS.

[CR31] MacPhail CL, Sayles JN, Cunningham W, Newman PA (2012). Perceptions of sexual risk compensation following posttrial HIV vaccine uptake among young South Africans. Qual Health Res.

[CR32] Marconi VC, Grandits G, Okulicz JF, Wortmann G, Ganesan A, Crum-Cianflone N, Polis M, Landrum M, Dolan MJ, Ahuja SK, Agan B, Kulkarni H (2011). Cumulative viral load and virologic decay patterns after antiretroviral therapy in HIV-infected subjects influence CD4 recovery and AIDS. PLoS ONE.

[CR33] Medlock J, Pandey A, Parpia AS, Tang A, Skrip LA, Galvani AP (2017). Effectiveness of UNAIDS targets and HIV vaccination across 127 countries. Proc Natl Acad Sci USA.

[CR34] Moodley N, Gray G, Bertram M (2016). Projected economic evaluation of the national implementation of a hypothetical HIV vaccination program among adolescents in South Africa, 2012. BMC Public Health.

[CR35] Moodley N, Gray G, Bertram M (2016). The case for adolescent HIV vaccination in South Africa: a cost-effectiveness analysis. Medicine.

[CR36] Mugglin C, Wandeler G, Estill J, Egger M, Bender N, Davies MA, Keiser O (2013). Retention in care of HIV-infected children from HIV test to start of antiretroviral therapy: systematic review. PLoS ONE.

[CR37] Ott MQ, Bärnighausen T, Tanser F, Lurie MN, Newell ML (2011). Age-gaps in sexual partnerships: seeing beyond ’sugar daddies’. AIDS.

[CR38] Pettifor A, Bekker LG, Hosek S, DiClemente R, Rosenberg M, Bull SS, Allison S, Delany-Moretlwe S, Kapogiannis BG, Cowan F (2013). Preventing HIV among young people: research priorities for the future. J Acquir Immune Defic Syndr.

[CR39] Phillips AN, Cambiano V, Nakagawa F, Ford D, Lundgren JD, Roset-Bahmanyar E, Roman F, Effelterre TV (2014). Potential future impact of a partially effective HIV vaccine in a southern African setting. PLoS ONE.

[CR40] Rehle T, Shisana O, Pillay V, Zuma K, Puren A, Parker W (2007). National HIV incidence measures-new insights into the South African epidemic. S Afr Med J.

[CR41] Robb ML, Rerks-Ngarm S, Nitayaphan S, Pitisuttithum P, Kaewkungwal J, Kunasol P, Khamboonruang C, Thongcharoen P, Morgan P, Benenson M, Paris RM, Chiu J, Adams E, Francis D, Gurunathan S, Tartaglia J, Gilbert P, Stablein D, Michael NL, Kim JH (2012). Risk behaviour and time as covariates for efficacy of the HIV vaccine regimen ALVAC-HIV (vCP1521) and AIDSVAX B/E: a post-hoc analysis of the Thai phase 3 efficacy trial RV 144. Lancet Infect Dis.

[CR42] Rubincam C, Newman PA, Atujuna M, Bekker LG (2018). ‘Why would you promote something that is less percent safer than a condom?’ Perspectives on partially effective HIV prevention technologies among key populations in South Africa. SAHARA J J Soc Asp HIV/AIDS.

[CR43] Russell ND, Marovich MA (2016). Pox-protein public private partnership program and upcoming HIV vaccine efficacy trials. Curr Opin HIV AIDS.

[CR44] Sayles JN, Macphail CL, Newman PA, Cunningham WE (2009). Future HIV vaccine acceptability among young adults in South Africa. Health Educ Behav.

[CR45] Selinger C, Bershteyn A, Dimitrov D, Hallett TB, Bekker LG, Rees H, Gray G (2017) Population-level impact and cost-effectiveness of an HIV vaccine in South Africa. In: CROI2017, Seattle, WA. http://www.croiconference.org/sessions/population-level-impact-and-cost-effectiveness-hiv-vaccine-south-africa. Accessed 1 Apr 2019 10.1016/j.vaccine.2019.02.073 PMC6684280 30890385

[CR46] Selinger C, Bershteyn A, Dimitrov DT, Adamson BJ, Revill P, Hallett TB, Phillips A, Bekker LG, Rees H, Gray G (2019). Targeting and vaccine durability are key for population-level impact and cost-effectiveness of a pox-protein HIV vaccine regimen in South Africa. Vaccine.

[CR47] Shisana O, Simbayi L (2002). Nelson Mandela/HSRC study of HIV/AIDS: South African national HIV prevalence, behavioural risks and mass media: household survey 2002.

[CR48] Shisana O, Rehle T, Simbayi L, Zuma K, Jooste S, Pillay-Van Wyk V, Mbelle N, Van Zyl J, Parker W, Zungu N, Pezi S, Implementation Team SABSSMIII (2010). South African national HIV prevalence, incidence, behaviour and communication survey 2008: a turning tide among teenagers?.

[CR49] Shisana O, Rehle T, Simbayi LC, Zuma K, Jooste S, Zungu N, Labadarios D, Onoya D, Wabiri N (2014) South African national HIV prevalence, incidence and behaviour survey 2012. HSRC Press, Cape Town. http://www.hsrc.ac.za/uploads/pageContent/4565/SABSSM IV LEO final.pdf. Accessed 1 Apr 2019 10.2989/16085906.2016.1153491 27002359

[CR50] Smith JA, Anderson SJ, Harris KL, McGillen JB, Lee E, Garnett GP, Hallett TB (2016). Maximising HIV prevention by balancing the opportunities of today with the promises of tomorrow: a modelling study. Lancet HIV.

[CR51] South African National AIDS Council (2016) South African HIV and TB investment case. http://sanac.org.za/wp-content/uploads/2016/03/1603-Investment-Case-Report-LowRes-18-Mar.pdf. Accessed 2017-05-10

[CR52] Statistics South Africa (2014) Mortality and causes of death in South Africa, 2012: findings from death notification. Statistics South Africa, Pretoria

[CR53] Unaids (2016) Global AIDS update 2016. http://www.unaids.org/sites/default/files/media_asset/global-AIDS-update-2016_en.pdf. Accessed 2017-05-09

[CR54] Unaids (2018) Global AIDS update 2018. http://www.unaids.org/sites/default/files/media_asset/unaids-data-2018_en.pdf. Accessed 2018-12-12

[CR55] UnitedNations (2016) World population prospects. Population Division, United Nations. http://esa.un.org/unpd/wpp/DVD/. Accessed 1 Apr 2019

[CR56] Voeten HACM, Vissers DCJ, Gregson S, Zaba B, White RG, de Vlas SJ, Habbema JDF (2009). Strong association between in-migration and HIV prevalence in Urban Sub-Saharan Africa. Sex Transm Dis.

