# Supplementary material for: The future of a partially effective HIV vaccine: assessing limitations at the population level
Source: Int J Public Health. 2019 Apr 13;64(6):957–64. doi: 10.1007/s00038-019-01234-z (PMC6614161; doi:10.1007/s00038-019-01234-z)
Supplement: Supplementary file 1 — Supplementary material 1 (pdf 618 KB) [file 38_2019_1234_MOESM1_ESM.pdf]

(will be inserted by the editor)

Supplementary Material for:
The future of a partially effective HIV vaccine: assessing limitations at the population level

blinded authors

Received: date / Accepted: date

blinded authors information

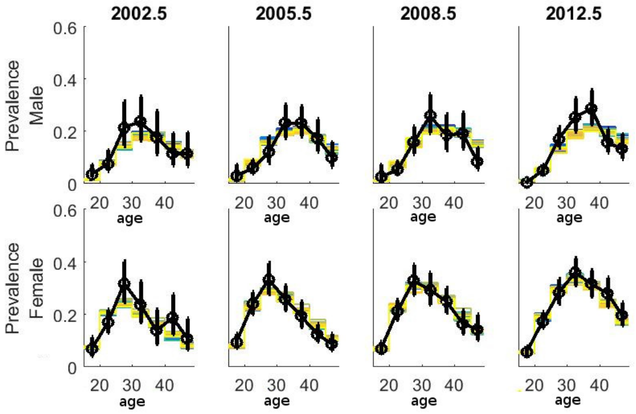

(a) Prevalence stratified by age (15-24, 25-29, 30-34, 35-39, 40-44, 45-49) and gender compared to HSRC survey data from 2002, 2005, 2008 and 2012.

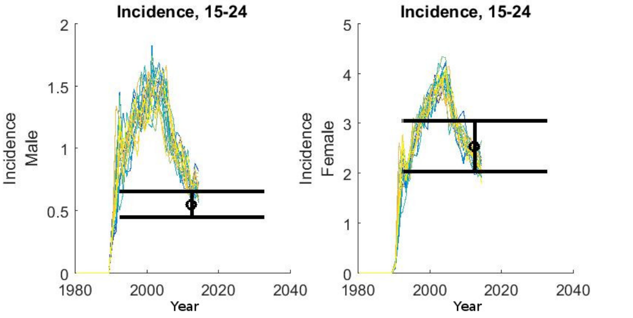

(b) Incidence age 15-24, stratified by gender

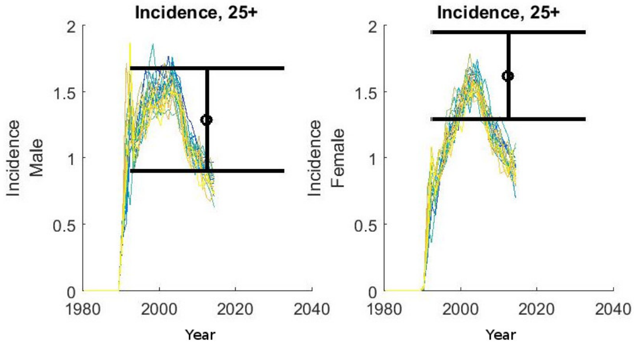

(c) Incidence age 25 and older, stratified by gender

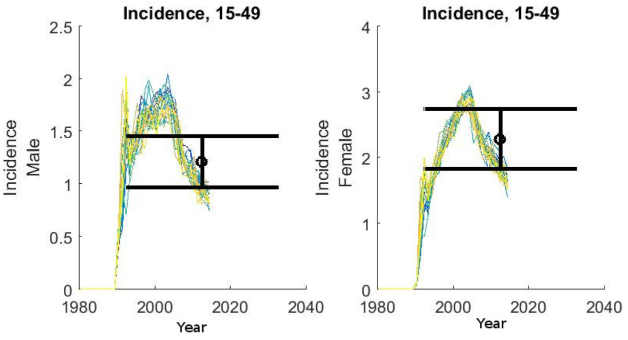

(d) Incidence age 15-49, stratified by gender

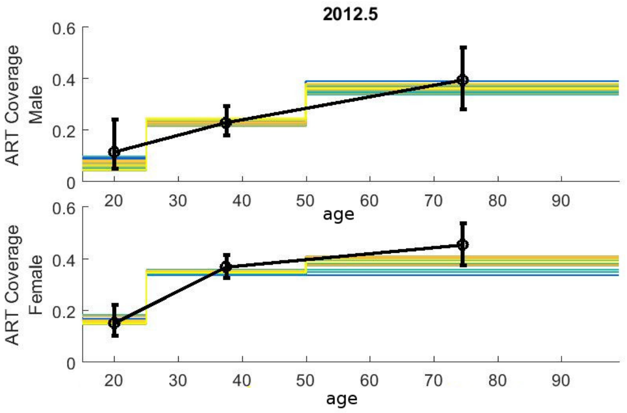

(e) ART coverage, stratified by age (15-24, 25-29, 30-34, 35-39, 40-44, 45-49) and gender

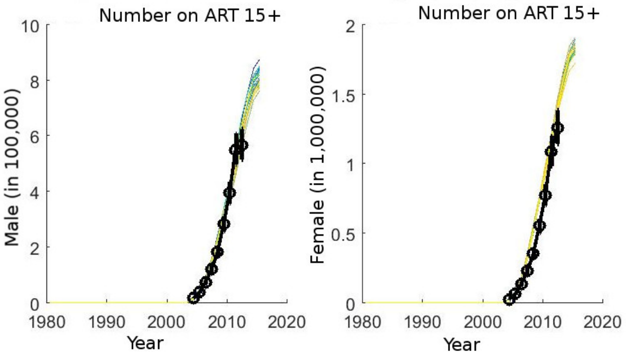

(f) Number on antiretroviral therapy (ART) stratified by gender

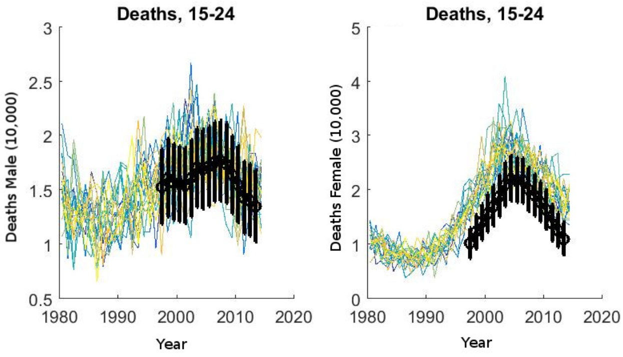

(g) Deaths (all cause mortality) by gender for age 15-24.

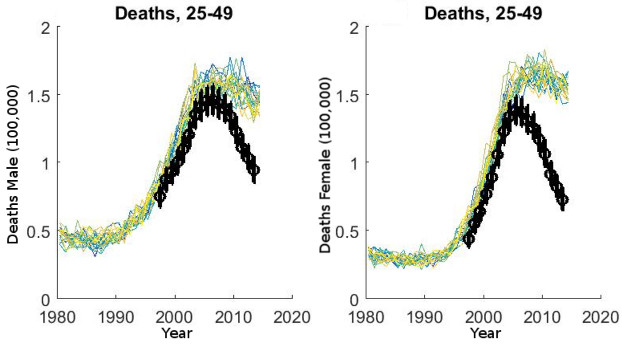

(h) Deaths (all cause mortality) by gender for age 25-49.

Fig. 1: Results of model calibration (trajectories in colors) to incidence, prevalence, ART coverage and all-cause mortality estimates [**? ? ? ? ? ?**] for South Africa (black).

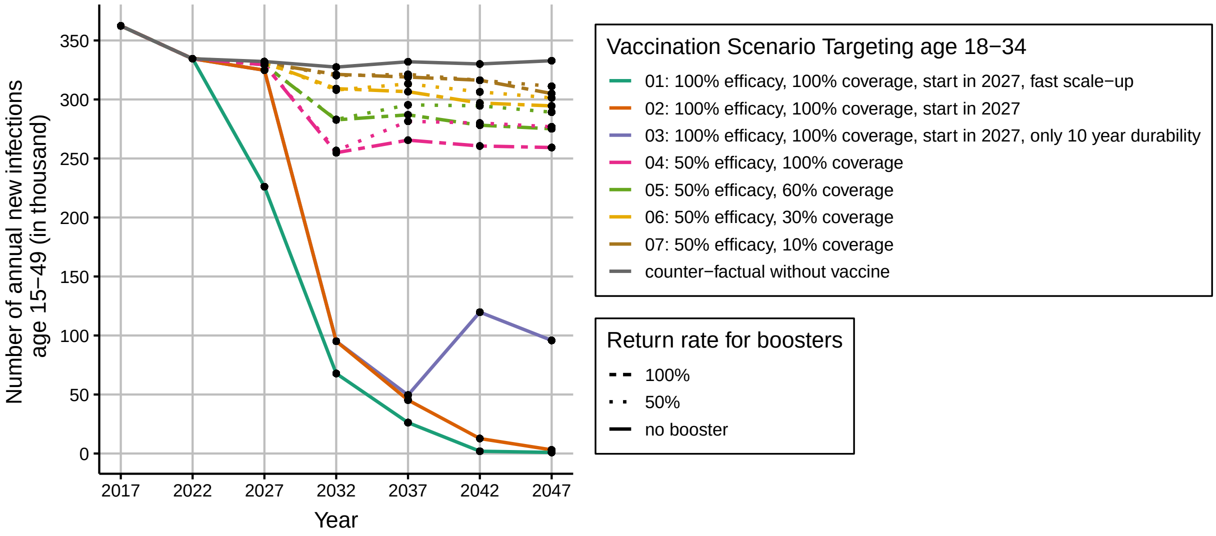

Fig. 2: Number of annual new infections (in thousand) under different vaccination scenarios. Note: the rise in annual new infection between 2037 and 2042 for scenario 3 stems from the fact that in 2037 vaccine efficacy is abruptly set to zero for those having received the vaccine ten year before during catch-up vaccination and that this cannot be compensated by cohort vaccination introduced in 2032. Similar conclusions hold for scenarios 4-7 where partial and waning efficacy for catch-up vaccines expires after five years, if booster vaccination is not maintained.

Parameter modified during model calibration	Parameter value (95% CI)
Base_Infectivity	0.000852 (0.00082-0.000885)
TRANSITORY_Formation_Rate_Constant	0.000911 (0.000872-0.000951)
INFORMAL_Formation_Rate_Constant	0.000744 (0.000703-0.000787)
MARITAL_Formation_Rate_Constant	6.1e-05 (5.9e-05-6.3e-05)
Male_To_Female_Relative_Infectivity_Multipliers	4.506283 (4.433338-4.574492)
Defaults_TRANSITORY_Condom_Usage_Probability_Mid	2006.163945 (2005.883396-2006.444411)
Defaults_TRANSITORY_Condom_Usage_Probability_Rate	2.428776 (2.297637-2.554955)
Defaults_TRANSITORY_Condom_Usage_Probability_Max	0.620835 (0.600207-0.641391)
Defaults_INFORMAL_Condom_Usage_Probability_Mid	1997.618152 (1996.72279-1998.576731)
Defaults_INFORMAL_Condom_Usage_Probability_Rate	0.779877 (0.663685-0.899579)
Defaults_INFORMAL_Condom_Usage_Probability_Max	0.345952 (0.328258-0.363927)
Defaults_MARITAL_Condom_Usage_Probability_Mid	1999.249259 (1998.546359-2000.029841)
Defaults_MARITAL_Condom_Usage_Probability_Rate	2.668629 (2.61344-2.721196)
Defaults_MARITAL_Condom_Usage_Probability_Max	0.242031 (0.231149-0.251669)
Initial_Distribution_Risk3	0.227233 (0.216862-0.237837)
Weighting_Matrix_RowMale_ColumnFemale_RiskAssortivity	0.352385 (0.312122-0.389567)
TRANSITORY_LOW_Prob_Extra_Relationship_Male	0.361095 (0.347754-0.375162)
TRANSITORY_MEDIUM_Prob_Extra_Relationship_Male	0.740209 (0.720398-0.759972)
TRANSITORY_LOW_Prob_Extra_Relationship_Female	0.162258 (0.151891-0.17433)
TRANSITORY_MEDIUM_Prob_Extra_Relationship_Female	0.445975 (0.419467-0.474465)
INFORMAL_LOW_Prob_Extra_Relationship_Male	0.422101 (0.399975-0.446544)
INFORMAL_LOW_Prob_Extra_Relationship_Female	0.134696 (0.120834-0.148558)
TRANSITORY_LOW_Max_Simultaneous_Relationships_Male	1.708727 (1.680612-1.7371)
TRANSITORY_LOW_Max_Simultaneous_Relationships_Female	1.708727 (1.679571-1.737707)
INFORMAL_LOW_Max_Simultaneous_Relationships_Male	1.211347 (1.157796-1.262011)
INFORMAL_LOW_Max_Simultaneous_Relationships_Female	1.211347 (1.161407-1.263891)
INFORMAL_MEDIUM_Prob_Extra_Relationship_Male	0.44122 (0.430161-0.452414)
INFORMAL_MEDIUM_Prob_Extra_Relationship_Female	0.172667 (0.157184-0.189508)
TRANSITORY_MEDIUM_Max_Simultaneous_Relationships_Male	2.610953 (2.543659-2.68037)
TRANSITORY_MEDIUM_Max_Simultaneous_Relationships_Female	2.610953 (2.541772-2.682377)
INFORMAL_MEDIUM_Max_Simultaneous_Relationships_Male	1.571329 (1.462857-1.67974)
INFORMAL_MEDIUM_Max_Simultaneous_Relationships_Female	1.571329 (1.462112-1.685857)
MARITAL_MEDIUM_Max_Simultaneous_Relationships_Male	0.945246 (0.930262-0.961531)
MARITAL_MEDIUM_Max_Simultaneous_Relationships_Female	0.945246 (0.930657-0.962727)

Table 1: Model parameters used during calibration process. We used the 50 best simulations to calculate the average and boot-strapped 95% confidence intervals. We refer to extensive online documentation (`http://idmod.org/docs/hiv/parameter-overview.html`) for further information on model parameters.

References

. Eaton JW, Bacar N, Bershteyn A, Cambiano V, Cori A, Dorrington RE, Fraser C, Gopalappa C, Hontelez JAC, Johnson LF, Klein DJ, Phillips AN, Pretorius C, Stover J, Rehle TM, Hallett TB (2015) Assessment of epidemic projections using recent HIV survey data in South Africa: a validation analysis of ten mathematical models of HIV epidemiology in the antiretroviral therapy era. Lancet Glob Health 3(10):e598–608, DOI 10.1016/S2214-109X(15)00080-7

. Rehle T, Shisana O, Pillay V, Zuma K, Puren A, Parker W (2007) National HIV incidence measures–new insights into the South African epidemic. S Afr Med J 97(3):194–9

. Shisana O, Simbayi L (2002) Nelson Mandela/HSRC study of HIV/AIDS: South African national HIV prevalence, behavioural risks and mass media: household survey 2002. HSRC Press

. Shisana O, Rehle T, Simbayi L, Zuma K, Jooste S, Pillay-Van Wyk V, Mbelle N, Van Zyl J, Parker W, Zungu N, Pezi S, SABSSM III Implementation Team (2010) South African National HIV Prevalence, Incidence, Behaviour and Communication Survey 2008: A Turning Tide Among Teenagers? HSRC Press, Cape Town

. Shisana O, Rehle T, LC S, Zuma K, Jooste S, N Z, Labadarios D, Onoya D, Wabiri N (2014) South African National HIV Prevalence, Incidence and Behaviour Survey 2012. HSRC Press, Cape Town, URL http://www.hsrc.ac.za/uploads/pageContent/4565/SABSSMIVLEOfinal.pdf

. Statistics South Africa (2014) Mortality and causes of death in South Africa, 2012: findings from death notification/Statistics South Africa.
